# Supplementary material for: WordCommentsAnalyzer: A windows software tool for qualitative research
Source: F1000Res. 2018 Sep 4;7:536. Originally published 2018 May 3. [Version 2] doi: 10.12688/f1000research.14819.2 (PMC6137411; doi:10.12688/f1000research.14819.2)
Supplement: Supplementary file 3 [file f1000research-7-17614-s0002.tgz › b2a79496-4ada-47c4-9682-a99ce867836d.zip!/Eight Iranian Health Journals/Salmand_Iranian Journal of Ageing _39.docx]

© Salmand_Iranian Journal of Ageing . This work is licensed under CC BY-NC 4.0 (https://creativecommons.org/licenses/by-nc/4.0/).

Volume & Issue: دوره 3 - شماره 3 و 4

Title: مطالعه تطبیقی موقعیت و جایگاه سالمندان در جوامع گذشته و معاصر

http://salmandj.uswr.ac.ir/article-1-96-fa.html

تغییرات اقتصادی-اجتماعی، همراه با پیشرفت علوم پزشکی موجب کاهش مرگ و میر، افزایش طول عمر و امید به زندگی طی قرون اخیر گردیده است که در نهایت منجر به رشد جمعیت سالمندان و سالخورده شدن جمعیت گردیده است. علیرغم افزایش تعداد و نسبت سالمندان کشور در چند دهه اخیر، موقعیت و جایگاه آنان به ویژه در شهرهای بزرگ در مقایسه با گذشته تضعیف شده و آن‌ها را با مشکلاتی مواجه ساخته است. در جوامع سنتی، سالمندان تمامی ابزارهای لازم برای اقتدار و سروری؛ به ویژه ثروت، قدرت، و حیثیت یا اعتبار اجتماعی را در اختیار داشته و با این ابزارها قادر به کسب منزلت اجتماعی برجسته و ایفای نقش‌های کلیدی در خانواده و جامعه بودند. اما تحولات ایجاد شده در عرصه‌های اجتماعی، اقتصادی، حقوقی، آموزشی، و خانوادگی در دوران جدید، سبب کاهش و نزول موقعیت و جایگاه سالمندان در مقایسه با جوامع گذشته گردیده است. این کاهش اقتدار و منزلت سالمندان در جوامع جدید و تغییر در نقش آن‌ها، سبب شکل‌گیری یک عقیده قالبی و باور عمومی گردید که سالمندان، موجوداتی ناتوانند که نمی‌توانند نقش فعالی در فرآیند توسعه جامعه ایفا نمایند. شاید گام اول برای پیدا کردن پاسخ‌هایی دقیق به پرسش‌هایی از قبیل این که آیا واقعا سالمندان نیرویی بی‌فایده و دور ریختنی‌اند؟ و چگونه می‌توان از توانایی‌های بالقوه و واقعی شان در توسعه جامعه بهره برد؟ شناسایی علل کاهش منزلت و جایگاه سالمندان در جوامع جدید باشد. در مجموع، صاحب‌نظران برخی از عوامل نظیر؛ سست‌شدن و تضعیف نقش سنت‌ها در تنظیم روابط اجتماعی و خانوادگی، افزایش نقش وسایل ارتباطی مکتوب و فن آوری‌های نوین ارتباطی در انتقال دانش‌ها، کاهش مشارکت سالمندان در فرآیند تولید اقتصادی، کاهش حجم خانواده و هسته‌ای شدن آن، کاهش کارکردهای نهاد خانواده، اشتغال زنان، شکاف نسل‌ها و ایجاد فاصله بین سالمندان و فرزندان و نوه‌هایشان، ارزشمند شدن جوانی و ظواهر فیزیکی و جسمی افراد در دنیای مدرن، و تغییر در معماری و فضای حیاتی منازل مسکونی را به عنوان مهم‌ترین علل کاهش جایگاه سالمندان در جوامع جدید برشمرده‌اند. با عنایت به چند بعدی بودن پدیده سالمندی، ارتقا منزلت و جایگاه سالمندان و احیای مجدد آن‌ها جهت ایفای نقش‌های مفید در فرآیند توسعه کشور، نیازمند تلاش و هماهنگی تمامی نهادها و سازمان‌های اجتماعی، اقتصادی، سیاسی، حقوقی، بهداشتی و درمانی می‌باشد.

Title: مقایسه تمرینات ذهنی، فیزیکی و ترکیبی بر تعادل ایستا و پویای سالمندان سالم

http://salmandj.uswr.ac.ir/article-1-97-fa.html

اهداف: پس از گذشتن از مرز 65 سالگی معمولا سالیانه 10 درصد سالمندان استقلال خود را در یک یا چند فعالیت روزانه از دست می دهند. دلیل این امر اغلب تغییرات پسرونده عصبی - عضلانی است که عامل مهمی در کاهش توانایی تعادل و راه رفتن در این گروه از افراد است. با توجه به آنچه بیان شد، هدف پژوهش حاضر مقایسه تمرینات ذهنی، فیزیکی و ترکیبی بر تعادل ایستا و پویای سالمندان سالم بود. مواد و روش‌ها: این تحقیق از نوع نیمه تجربی با طرح پیش آزمون - پس آزمون با سه گروه آزمایشی است. نمونه آماری این تحقیق را 60 نفر از سالمندان شهرکرد با دامنه سنی 60-70 سال تشکیل دادند، که به صورت تصادفی به سه گروه آزمایشی تقسیم شدند. در این تحقیق جهت اندازه گیری تعادل ایستای سالمندان از آزمون تعادلی لک لک (ایستادن بر روی یک پا) و جهت اندازه گیری تعادل پویای سالمندان از آزمون تعادل پویای SEBT استفاده شد. پس از 8 جلسه تمرین از هر یک از گروه ها پس آزمون که مشابه با مرحله پیش آزمون بود به عمل آمد. جهت تجزیه و تحلیل داده ها از آزمون تحلیل واریانس یک راهه و آزمون تعقیبی توکی و به منظور اطمینان از همسانی واریانس ها در مرحله پیش آزمون از آزمون همسانی واریانس ها (آزمون لون) در سطح )P<0/05( استفاده شد. یافته‌ها: یافته های آماری پژوهش در سه روش تمرینی (ذهنی، فیزیکی و ترکیبی) در تعادل ایستا و پویا نشان داد که بین عملکرد گروه ها در مرحله پس آزمون تفاوت معنی داری وجود دارد. آزمون تعقیبی توکی نیز نشان داد بین گروه ترکیبی با دو گروه دیگر تفاوت معنی داری وجود دارد )P<0/001( و گروهی که به صورت ترکیبی (تمرین ذهنی - فیزیکی) تمرین کرده بودند نسبت به دو گروه دیگر عملکرد بهتری داشتند. همچنین عملکرد گروه تمرین فیزیکی نیز بهتر از گروه تمرین ذهنی بود (P<0/029). نتیجه‌گیری: با توجه به یافته های تحقیق حاضر که حاکی از پیشرفت گروه تمرین ترکیبی نسبت به دو گروه دیگر است، می توان نتیجه گیری کرد که برای افزایش قدرت عضلانی اندام های تحتانی و بهبود تعادل در سالمندان با استفاده از تمرینات قدرتی ویژه مربیان و مسوولین ذیربط می توانند از این روش تمرینی در برنامه های درمانی خود برای سالمندان بهره گیرند.

Title: تاثیر یک دوره تمرین قدرتی بر برخی پارامترهای کینماتیکی راه رفتن زنان سالمند سالم

http://salmandj.uswr.ac.ir/article-1-98-fa.html

اهداف: این تحقیق با هدف بررسی اثر حاصل از تمرینات قدرتی بر برخی پارامترهای کینماتیکی راه رفتن (طول گام، ریتم گام برداری و سرعت راه رفتن) در زنان سالمند انجام گرفت. مواد و روش‌ها: 24 زن سالمند سالم با میانگین سنی 61/53±2/84) سال)، قد 157/1±5/5) سانتی متر)، وزن 69/13±7/6) کیلوگرم) و BMI (28/1±3/6 کیلوگرم بر مترمربع) در این تحقیق شرکت کردند و بصورت تصادفی به دو گروه مساوی کنترل و آزمایش تقسیم شدند. قدرت اندام تحتانی آزدمودنیها با استفاده از آزمون پرس پا اندازه گیری شد. با فیلم برداری و استفاده از نرم افزارهای 3DMax، پریمایر و فتوشاپ سرعت راه رفتن، ریتم راه رفتن و طول یک گام قبل و بعد از دوره تمرینی اندازه گیری شد. گروه کنترل فعالیت عادی روزمره را دنبال کردند در حالی که گروه آزمایش به مدت هشت هفته، سه جلسه در هفته به تمرینات قدرتی برای اندام تحتانی عضلات نگه دارنده بدن پرداختند. از تست تی همبسته برای بررسی تفاوتهای درون گروهی و از تست تی مستقل برای تفاوت بین گروهی در سطح معناداری 0/05 استفاده شد. یافته‌ها: در گروه کنترل، هیچ یک از پارامترهای کینماتیکی اندازه گیری شده و قدرت اندام تحتانی، تغییری ایجاد نشد. در حالی که در گروه آزمایش، به استثنا ریتم راه رفتن، افزایش معناداری در اندازه های طول گام، سرعت راه رفتن و قدرت در اندام تحتانی مشاهده شد. در مقایسه بین دو گروه به استثنا ریتم راه رفتن، طول گام، سرعت راه رفتن و قدرت اندام تحتانی در گروه آزمایش افزایش معناداری نسبت به گروه کنترل نشان داد. نتیجه‌گیری: نتایج این مطالعه تاثیر تمرین قدرتی و افزایش قدرت عضلانی در اندام تحتانی و عضلات نگه دارنده بدن را در افزایش اندازه طول گام و سرعت راه رفتن در زنان سالمند سالم تایید نمود.

Title: بررسی تأثیر نوع سطح اتکا و نوع موضوع توجه بر تعادل مردان سالمند با سابقه افتادن

http://salmandj.uswr.ac.ir/article-1-99-fa.html

اهداف: دستورات کلامی مربوط به نحوه هدایت توجه به حرکت یا پیامدهای آن بر اجرا و یادگیری مهارتهای حرکتی اثرگذار است. این اثرگذاری با افزایش دشواری تکلیف الگویی متفاوت می یابد. هدف بررسی حاضر آن است که اثرات نوع سطح اتکا را بر میزان تاثیرگذاری موضع توجه بیازماید. مواد و روش‌ها: 28 نفر سالمند 65 سال و بالاتر که در یک سال گذشته حداقل سابقه یکبار افتادن را داشته و ملاکهای ورود به پژوهش را دارا بودند، از فرهنگسرای سالمند بطور نمونه گیری در دسترس انتخاب شده و به محل آزمایش واقع در بخش کاردرمانی هلال احمر جمهوری اسلامی ایران انتقال داده شدند. در این پژوهش که بصورت طرح دو عاملی درون گروهی با اندازه گیری های تکرار شونده انجام گرفت، پس از اخذ رضایت نامه کتبی، هر شرکت کننده به ترتیب در 6 وضعیت آزمون یعنی ابتدا درسطح اتکای ثابت و سپس سطح اتکای متحرک و نیز در ابتدا حالت پایه ای (بدون موضع توجه)، سپس موضع درونی توجه و در آخر موضع بیرونی توجه قرار گرفته و نمره تعادل آنها که توسط دستگاه تعادل سنج نوروکام حاصل میشد، ثبت گردید. یافته‌ها: افزایش دشواری تکلیف یعنی تغییر سطح اتکا از ثابت به متحرک بطور کلی سطح عملکرد را پایین آورد. اما بیشترین میزان افت عملکرد در حالت موضع درونی توجه / سطح اتکای متحرک و کمترین میزان افت عملکرد در حالت موضع توجه بیرونی/سطح اتکای متحرک دیده شد. با آزمون ANOVA دوطرفه مشخص شد که نوع سطح اتکا و نوع موضع توجه اثر متقابل معناداری با همدیگر دارند (P<0/001). نتیجه‌گیری: بخشی از این یافته ها، نتایج مطالعات پیشین را تایید می نمود. نتیجه جالب توجه آن است که ارایه نکردن دستور کلامی نسبت به ارایه دستور کلامی موضع توجه درونی بهتر است. میزان اثر ارایه دستور کلامی موضع توجه بیرونی در شرایط دشواری تکلیف، بارزتر بوده و اثرات مثبت بیشتری نسبت به حالتهای دیگر موضع توجه، بر روی کیفیت حفظ تعال می گذارد.

Title: بررسی روایی و پایایی آزمون پیشنهادی پس (PASS) در ارزیابی مراقبت از خود بیماران پارکینسون

http://salmandj.uswr.ac.ir/article-1-100-fa.html

اهداف: بیماری پارکینسون یک بیماری پیشرونده می باشد که تلاش در جهت حفظ استقلال در انجام فعالیت های روزمره زندگی و مهارتهای مراقبت از خود تا حد امکان، برای این بیماران بعنوان مهمترین مساله در نظر گرفته می شود. آزمون مهارتهای مراقبت از خود (PASS)  از سه بخش فعالیتهای روزمره زندگی (A.D.L.)، کاربرد ابزار در فعالیتهای روزمره زندگی I.A.D.L)) و تحرک عملکردی در زندگی روزمره (Mobility)  تشکیل شده است. روایی و پایایی این آزمون تنها در طیف بسیار محدودی از ناتوانایی ها مورد مطالعه قرار گرفته است و بر روی بیماران پارکینسون اصلا انجام نشده است. هدف از انجام این مطالعه، بررسی روایی و پایایی آزمون مهارتهای مراقبت از خود (PASS)  در بیماران پارکینسون می باشد. مواد و روش‌ها: آزمون PASS  جهت تعیین روایی در ابتدا ترجمه شد، آزمون به همراه پرسشنامه تدوین شده جهت این کار در اختیار 5 نفر کارشناس که همگی عضو هیات علمی کاردرمانی بودند قرار گرفت. جهت تعیین پایایی نیز از مراکز توانبخشی 5 منطقه جغرافیایی شهر تهران به تعداد 50 نمونه در رده های سنی45  تا 85سالگی بصورت موارد در دسترس انتخاب گردید. پس از آن آزمون مورد نظر با استفاده از دستورالعمل PASS  در دو مرحله از نظر استقلال، ایمنی و نتیجه بررسی گردید. یافته‌ها: توافق میان نظر کارشناسان در خصوص روایی آزمون PASS برای بیماران پارکینسون از نظر استقلال (X2=5/83, DF=4)، ایمنی (X2=5/44, DF=4) و نتیجه (X2=2/45, DF=4) با اطمینان بیش از %95  وجود داشت. همچنین همبستگی آزمون-بازآزمون تست مهارتهای مراقبت از خود (PASS)  برای بیماران پارکینسون از نظر استقلال با ضریب همبستگی r=0/93، ایمنی با ضریب همبستگی r=0/91  و نتیجه با ضریب همبستگی r=0/91، در سطح  (P=0/01) معنی دار بود که نشان دهنده پایایی بالای این آزمون است. نتیجه گیری: نتایج پژوهش حاضر نشان می دهد که این آزمون برای بیماران پارکینسون از پایایی و روایی بالایی برخوردار است. این یافته ها از این نظر قابل انتظار بود که در این بیماران آزمون عملکردی چندان تغییراتی نداشته و ماده های آزمون متناسب با اهداف درمانی بودند و آشنایی کافی آزمودنی ها با مهارتهای مربوطه و مرحله بندی در نمره دهی استاندارد آزمون نیز نشان دهنده پایایی و روایی قابل قبول آزمون بودند. بنابراین در کل می توان نتیجه گرفت که این ارزیابی می تواند بعنوان ابزاری مناسب در اختیار متخصصان قرار بگیرد.

Title: مقایسه نتایج حاصل از جراحی باز و لاپاروسکوپی در سالمندان

http://salmandj.uswr.ac.ir/article-1-101-fa.html

اهداف: با افزایش سن شیوع سنگ صفراوی نیز رو به ازدیاد است و سنگهای صفراوی شایعترین اندیکاسیون جراحی شکمی در سالمندان میباشد. از طرفی سالمندان به خاطر بیماریهای همراه بیشتر در معرض عوارض جراحی میباشند. جراحی کوله سیستکتومی لاپاراسکوپی استاندارد طلایی در درمان سنگهای صفراوی است. هدف از این مطالعه بررسی اثرات و فواید کوله سیستکتومی لاپاراسکوپیک در سالمندان و مقایسه آن با جراحی باز می باشد. مواد و روش‌ها: در این مطالعه آینده نگر از تیر8313 تا اسفند1386 کلیه بیماران سالمند (سن(60< که دارای نمره ASA<3 (American Society of Anesthesiologists score)  و نیاز به کوله سیستکتومی داشتند وارد این مطالعه میشدند و به 2 گروه الف: کوله سیستکتومی باز و گروه ب: کوله سیستکتومی لاپاراسکوپی تفکیک و پیامدهای حاصل از عمل جراحی شامل مورتالیتی – موربیدیتی - طول مدت بستری و مدت زمان عمل جراحی بین دو روش مقایسه می شدند. یافته‌ها: از تعداد311  بیمار کوله سیستکتومی شده206  نفر زن و105  نفر مرد بودند. میانگین سنی بیماران71/4  سال بود 157 بیمار تحت عمل باز و  154 بیمار نیز تحت کوله سیستکتومی لاپاراسکوپی قرار گرفتند 18 .بیمار ASA score=I  و 193  بیمار ASA score=II  و 100 بیمار  ASA score=III داشتند.میزان مورتالیتی و موربیدیتی در گروه ب به ترتیب %2  و صفر و مدت اقامت در بیمارستان نیز 1/1  روز که نسبت به گروه الف کمتر و از نظر آماری نیز معنی دار بود. نتیجه گیری: کوله سیستکتومی لاپاراسکوپیک یک روش انتخابی برای بیماران سالمند است و نتایج عمل کوله سیستکتومی لاپاراسکوپیک نیز نسبت به عمل باز بهتر است.

Title: ارتباط نمره شاخص بارتل در بدو پذیرش با نتایج کلینیکی بیماران سالمند هنگام ترخیص

http://salmandj.uswr.ac.ir/article-1-102-fa.html

اهداف: هدف این مطالعه ارزیابی ارتباط بین شاخص بارتل و برون ده کلینیکی در بیماران سالمند بالای 60 سال در خلال پیگیری 4  هفته بعد از بستری اولیه این بیماران بود. مواد و روش‌ها: این پژوهش یک مطالعه طولی آینده نگر بود 50 مرد و 56  زن سالمند بیمار که در بخش اورژانس پذیرش شده بودند و معیارهای ورود (داشتن سن بالای 60 سال و رضایت از شرکت در پژوهش) را داشتند، مورد مطالعه قرار گرفتند. تمام بیماران مورد بررسی بعد از 4 هفته از بستری اولیه مورد ارزیابی قرار گرفتند. یافته‌ها: در پیگیری برون ده مشخص شد %81/1 سالمندان بیمار پس از چهار هفته مرخص، %8/5 آنان کماکان بستری و %10/4  از کل بیماران فوت کرده اند. همچنین نتایج نشان داد بازافزایش یک واحد به نمره کل بارتل شاس ترخیص در مقایسه با مرگ %20  افزایش پیدا می کند. همچنین با افزایش یکسال به سن بیماران از نمره کل بارتل آنان مقدار 0/23 واحد کم می شود. نتیجه‌گیری: نتایج این مطالعه نشان می دهد که بیماران سالمند دارای سن بالاتر یا نمره کلی بارتل بدو مراجعه پایین تر در وضعیت خطرناک‌تری بوده و احتیاج به توجه و مراقبت ویژه دارند.

Title: تحلیل تجارب بیماران سالمند ایرانی از سکته قلبی

http://salmandj.uswr.ac.ir/article-1-103-fa.html

اهداف:  آگاهی از تجارب سالمندان مبتلا به سکته قلبی می تواند به تعیین نیازها، مشکلات، و نیز بهبود کیفیت زندگی آنها کمک کند، اما اطلاعات اندکی درباره تجارب آنان از سکته قلبی وجود دارد. در پژوهش حاضر، تجربه بیماران سالمند از سکته قلبی و رویکرد مقابله با بیماری مورد مطالعه قرار گرفته است. مواد و روش‌ها: این مقاله بخشی از مطالعه بزرگتری است که با رویکرد گراندد تئوری انجام شده است. با هشت بیمار مبتلا به سکته قلبی سنین 65 سال به بالا که به صورت نمونه گیری هدفمند و نظری انتخاب شدند، مصاحبه گردید. مصاحبه نیمه ساختار یافته روش اصلی جمع آوری داده ها بوده و تا زمان اشباع داده ها ادامه یافت. داده ها بطور همزمان با جمع آوری اطلاعات با استفاده از روش اشتراوس و کوربین تحلیل گردید. اعتبار داده ها بر اساس معیارهای پیشنهادی لیکنولن و گوبا تایید شد. یافته‌ها: تجارب سالمندان از سکته قلبی در دو درونمایه زندگی در سایه ترس، زندگی با محدودیت طبقه بندی شده اند و راهبردهای مقابله‌ای تحت عنوان بازگشت به زندگی فعال نام گذاری شده است. درونمایه زندگی در سایه ترس دارای زیر طبقات ترس از مرگ و سکته مجدد، ترس از ناتوانی جسمی، و ترس از وابسته شدن به دیگران می باشد .درونمایه زندگی با محدودیت شامل زیر طبقات احساس بی قدرتی جسمانی، علایم جسمی، و تمکین از دستورات/خانواده می باشد. درونمایه بازگشت به زندگی فعال شامل تفکر مثبت و خود اتکایی می باشد. نتیجه‌گیری: سالمندان مبتلا به سکته قلبی به دلیل تهدید ناگهانی سلامتی و زندگی، تنش های شدید و محدودیت های زیادی را تجربه می کنند. اتخاذ رویکردهای مثبت مقابله ای می تواند بازگشت به زندگی طبیعی را تسهیل نموده و کیفیت زندگی آنان را بهبود بخشد.

Title: کیفیت زندگی سالمندان منطقه 2 تهران

http://salmandj.uswr.ac.ir/article-1-104-fa.html

اهداف: جمعیت جهان بسرعت در حال سالمند شدن است امروزه در اکثر جوامع، سالمندی به عنوان یک مسئله مهم مطرح است. سالمندی فرآیند طبیعی در زندگی انسان و حاصل فرسایش تدریجی ارگانهای حیاتی است. عوامل فردی مانند سن، جنس، نژاد و ویژگی‌های اجتماعی و فرهنگی و اقتصادی در زمان و میزان آن تاثیر گذارند. همزمان با افزایش کمیت سالمندان توجه به کیفیت زندگی سالمندان و شناسایی عوامل موثر بر آن جهت سیاستگزاریهای جامع و صحیح برای این قشر از افراد جامعه از اهمیت زیادی برخوردار است. در این مقاله به بررسی وضعیت کیفیت زندگی سالمندان منطقه 2 تهران پرداخته شده است. مواد و روش‌ها: مطالعه حاضر مطالعه ای مقطعی و از نوع تحلیلی می باشد که اطلاعات آن با استفاده پرسشنامه های کیفیت زندگی مخصوص سالمندان (لیپاد) و چک لیست متغیرهای زمینه ای در یک نمونه 100 نفری از سالمندان منطقه 2 تهران جمع آوری شده است و به منظور تجزیه و تحلیل اطلاعات حاصله از آزمونهای کولموگروف - اسمیرنوف (K-S)، آنالیز واریانس یک طرفه (One way Anova) و آزمون تی (T.Test) استفاده شد. یافته‌ها: از جمعیت 100 نفری این تحقیق 70 نفر را مردان و 30 نفر را زنان سالمند تشکیل می دادند. اکثریت واحدهای مورد پژوهش  61) درصد) در دامنه سنی 60-70 سال بودند، میانگین سنی برای مردان حدود70  سال و برای زنان حدود 69 سال بود. بین مردان و زنان سالمند در تمام ابعاد کیفیت زندگی اختلاف آماری معناداری وجود داشت (P<0/05) و در تمامی ابعاد کیفیت زندگی مردان سالمند بالاتر بود. افراد متاهل و با تحصیلات بالاتر و دارای مسکن شخصی از کیفیت زندگی بالاتری برخوردار بودند. نتیجه‌گیری: عوامل مختلفی بر کیفیت زندگی سالمندان تاثیرگذار می باشند که شناخت این عوامل جهت برنامه ریزی بهتر در مورد سالمندان و توانمندسازی آنان از اهمیت خاصی برخوردار است.

Title: ارزیابی میزان ناتوانی در سالمندان شهر تهران (سال 1386)

http://salmandj.uswr.ac.ir/article-1-105-fa.html

اهداف: این مطالعه با هدف ارزیابی میزان ناتوانی در سالمندان شهر تهران انجام شده است. این تحقیق تلاش دارد با شناسایی و ارزیابی ناتوانی سالمندان در حوزه های مختلف با استفاده از ابزار WHODAS II سیاستگذاران و برنامه ریزان امور سالمندی را در تعیین خدمات موردنیاز سالمندان کمک کند. مواد و روش‌ها: این مطالعه به روش توصیفی و تحلیلی انجام شده است. اطلاعات با استفاده از پرسشنامه WHODAS II نسخه 36 سوالی بدون احتساب روز که توسط پرسشگر و با مصاحبه تکمیل می شد، جمع آوری گردید. نمرات خام کسب شده بر اساس الگوریتم سازمان جهانی بهداشت به امتیاز 0-100 تبدیل گردید. تعداد 600 نفر از افراد 60 ساله و بالاتر شهر تهران در این پژوهش با مراجعه به منزلشان مورد مصاحبه قرار گرفتند. روش انتخاب مصاحبه شوندگان، طبقه بندی خوشه ای بود. داده های حاصل با استفاده از آزمون های آماری خی دو، من ویتنی، کروسکال والیس و ضریب همبستگی مورد تجزیه و تحلیل قرار گرفت. یافته‌ها: میانگین نمرات ناتوانی سالمندان شهر تهران 33.3 با انحراف معیار 25/9 بود. از این میان نمرات ناتوانی برای زنان 33/6 با انحراف معیار 23/8 و برای مردان 33/0 با انحراف معیار 28/1 بود. میانگین نمرات ناتوانی سالمندان شهر تهران در میان مردان و زنان اختلاف معناداری نداشت (P>0/05). و میانگین نمرات ناتوانی سالمندان شهر تهران با افزایش سن افزایش پیدا می کرد (P>0/05) بیشترین ناتوانی آنان در حوزه های مورد بررسی مربوط به تحرک و جابجایی، انجام وظایف خانه و خانواده و مشارکت در جامعه بود. نتیجه‌گیری: نتایج این پژوهش نشان داد که میانگین نمرات ناتوانی سالمندان شهر تهران از میزان بالایی به ویژه در تحرک و جابجایی، انجام وظایف خانه و خانواده و مشارکت در جامعه برخوردار است. بنابراین لازم است توجه بیشتری به بیماری ها، اختلال ها و آسیب هایی که منجر به ناتوانی در میان سالمندان می شود از طریق اقدامات پیشگیرانه، درمان بموقع و موثر و توانبخشی بهنگام آنها داشته و در جهت طراحی و تامین وسایل کمکی موردنیاز و مناسب سازی محیط های داخل منزل و محیط های بیرونی نیز عمل نمود.
